# Supramolecular soft matter in traditional Chinese medicine: multiscale structure–function evidence, critical limitations, and translational challenges

**DOI:** 10.3389/fphar.2026.1945675

**Published:** 2026-10-21

**Authors:** Tianning Li, Baoguang Huang, Xiangyu Wang, Jiacheng Zhang, Yutian Zhu, Siqi Guan, Jingshang Wang

**Affiliations:** 1 Department of Traditional Chinese Medicine, Beijing Obstetrics and Gynecology Hospital, Capital Medical University, Beijing Maternal and Child Health Care Hospital, Beijing, China; 2 Department of Traditional Chinese Medicine, Peking University Third Hospital, Beijing, China

**Keywords:** limits of evidence, physicochemical determinants, quality by design, structure –function relationship, supramolecular self-assembly, traditional Chinese medicine-derived supramolecular soft matter

## Abstract

Plant metabolites used in traditional Chinese medicine (TCM) can form supramolecular assemblies in decoctions or in formulations prepared from purified molecules. These experimental settings support different conclusions. This narrative review examines TCM-derived supramolecular soft matter (TCM-derived SSM) through relationships among molecular interactions, assembly structure, physicochemical properties, and biological responses. It distinguishes assemblies isolated from decoctions, recombined extracts, formulations prepared from purified metabolites, and mixtures of defined metabolites with extracts. Micelles, vesicles, nanoparticles (NPs), nanofibers (NFs), and supramolecular gels provide examples, while carbonized products and covalently crosslinked carriers are discussed as related materials with distinct formation mechanisms. Selected studies associate morphology, surface chemistry, or deformability with altered delivery and preclinical activity. However, the presence of particles, successful reconstitution, and improved efficacy do not individually establish that an assembly is necessary for the effect of a complete preparation. Our contribution is an evidence-oriented comparison of preparation routes, structural measurements, biological comparisons, and the limits of inference across these preparations. We also examine artificial intelligence (AI)-assisted modeling and candidate critical quality attributes (CQAs) within a Quality by Design (QbD) perspective. Predictive design and assembly-based quality control are presented as research prospects that require reproducible measurements, appropriate controls, and validation across batches and biological settings. Self-assembly is a general physicochemical phenomenon; TCM provides a context for studying defined botanical drug combinations and preparation processes rather than evidence of properties unique to one medical tradition. We position this physical-state perspective within the established literature on synergy, antagonism and pharmacokinetic interactions in botanical mixtures.

## 1 Introduction

Traditional Chinese medicine (TCM) provides examples of botanical drug combinations in which extraction, heating, mixing, and storage may change both chemical composition and physical state. Plant metabolites are established sources of pharmacologically active molecules (Ma et al., 2020; Newman and Cragg, 2020), while studies of selected preparations have also identified colloidal or supramolecular fractions associated with biological activity (Zhou et al., 2014; Liang et al., 2023; Chen et al., 2026). These observations motivate investigation of assemblies as possible contributors to formulation behavior and efficacy. They do not establish that every preparation contains the same structures, that assemblies account for the whole therapeutic effect, or that self-assembly is specific to TCM.

Here, TCM-derived supramolecular soft matter (TCM-derived SSM) denotes molecular assemblies involving plant metabolites or botanical macromolecules whose organization depends substantially on non-covalent interactions. The structures discussed include micelles, vesicles, nanoparticles (NPs), nanofibers (NFs), and supramolecular gels. The term is an organizing description, not a claim that all botanical drug preparations form one material class with uniform properties. Dynamic exchange, deformability, and responsiveness require experimental characterization in each preparation. Carbonized products, chemically modified metabolites, and covalently crosslinked polymer carriers are distinguished from reversible metabolite assemblies and are retained only where they clarify the scope or provide a relevant delivery comparison. Carrier-free refers to formulations without a separate carrier matrix; it is not used for every material containing a plant metabolite.

Recent reviews already cover self-assembly mechanisms, cancer applications, functional biomaterials, decoction efficacy, and compatibility interpretation (Huang et al., 2023; Wang and Wang, 2026; Wang et al., 2026b). Accordingly, this review does not claim that the occurrence of self-assembly or its biomedical use is a new concept. Compared with these reviews, the present article makes three specific contributions. First, it classifies each example by the material actually tested, separating assemblies isolated from decoctions, recombined extracts, formulations prepared from purified metabolites, semi-defined mixtures, and hybrid or carbonized comparators (Table 1). Second, it evaluates which structural measurements and biological comparisons support a structure-function interpretation and where that inference stops; Section 3.4 and Table 2 apply this analysis to the identification of assembled species in crude preparations. Third, it states the evidence still needed before such interpretations can inform quality control or predictive design, including the controls, reporting items, and validation steps summarized in Section 7. This approach complements application-oriented summaries by evaluating the strength and scope of the connections among structure, properties, and function.

**TABLE 1:** Selected studies of plant-metabolite assemblies and related formulations, organized by preparation source, observed properties, biological findings, and limits of inference. Related hybrid materials are identified separately from reversible metabolite self-assemblies.

Material studied	Preparation source	Proposed interactions or formation	Preparation examined	Observed structure	Variable examined	Biological comparison	Supported finding and main limitation	References
Oleanolic acid (OA)	Engineered	Solvent-dependent assembly	Purified OA formulated under different aqueous ethanol conditions	Rigid NPs and flexible nanogels	Mechanical response and transport	Cellular uptake, tissue penetration and antitumor activity	Different OA formulations show different transport and activity; mechanics can covary with hydration and packing	Li et al., 2021
Scutellariae Radix and Coptidis Rhizoma extracts	Extract-recombined; separate defined model	Heat-associated reorganization; a purified baicalin-BBR pair was also studied	Scutellariae Radix and Coptidis Rhizoma each refluxed in 8 volumes of water for 15 min and freeze-dried; redissolved extracts mixed 1:1, with or without heating at 80 °C; separate purified-metabolite experiments (Table 3)	Fibrous and spherical structures	Heating, morphology and bacterial species	Activity against *S. aureus* and *B. subtilis*	Antibacterial preference differed by species. Recombined extracts are not direct samples of a fresh codecoction; purified-model mechanisms require separate attribution	Wei et al., 2025
BBR with flavonoid glycosides	Engineered	Association of defined BBR-flavonoid pairs	Purified baicalin-BBR and wogonoside-BBR formulations	NPs and NFs	Molecular partner, surface presentation and morphology	Antibacterial and biofilm assays	NP surface groups were associated with stronger bacterial affinity. Different partner molecules confound a shape-only causal interpretation	Li et al., 2019b
Ginsenoside-based insulin formulation	Engineered	Amphiphilic association and insulin incorporation	Film hydration, sonication and purification of a defined formulation	Micellar NPs	Membrane interaction and transport	Transdermal insulin delivery	Supports transdermal insulin delivery; membrane interactions should be assessed alongside insulin exposure and host-tissue safety	Zou et al., 2021
Penta-O-galloyl-D-glucose (PGG) epimers	Engineered	Hydrogen bonding and aromatic interactions	Defined glucose epimers examined for gelation in water	Supramolecular hydrogel	Stereochemical configuration	Macrophage responses and wound healing	Different epimers show different gelation. Configuration can affect both assembly and biological interactions, limiting attribution to the network alone	Lu et al., 2024
Naringenin-derived carbon dots in oxidized dextran	Engineered hybrid; carbonized material	Carbonization followed by dynamic covalent Schiff-base crosslinking	Thermally prepared carbon dots incorporated into a polymer hydrogel	Carbon dots and hydrogel	pH-sensitive network transformation	Antitumor treatment and release	A responsive composite is supported; its carbonized identity and polymer matrix preclude classification as unmodified naringenin self-assembly	Yan et al., 2026
GP-682 saponin	Engineered	Amphiphilic micelle formation	Defined saponin dispersed by solvent exchange and dialysis	Micelles	Host-cell permeability and lung exposure	Adjunct to separately administered levofloxacin in infection models	Host epithelial permeability and pulmonary antibiotic delivery improved. This is not evidence of bacterial membrane permeabilization or micellar antibiotic encapsulation	Zhang et al., 2020
Curcumin and iron oxide in GelMA	Engineered hybrid	Photocrosslinking of a gelatin methacryloyl matrix	Microfluidic fabrication of curcumin/iron oxide-containing hydrogel microspheres	Composite microspheres	Near-infrared irradiation and release	Preclinical endometriosis treatment	Supports a carrier-based photothermal formulation; matrix, iron oxide and irradiation effects cannot be assigned to curcumin self-assembly alone	Liu et al., 2024a
Glycyrrhizic acid (GA) and oxymatrine	Engineered	Ionic association and subsequent micellization	Defined ionic-liquid-derived formulation carrying a signal peptide	Nanomicelles	Skin-lipid interactions	Peptide penetration and anti-photoaging evaluation	Supports formulation-dependent skin transport; exposure and barrier effects require separate evaluation before generalizing to other preparations	Wang et al., 2025e
Ma-Xing-Shi-Gan-Tang	Decoction-derived	Colloidal fractions recovered after aqueous decoction	Decoction of Ephedrae Herba, honey-processed Glycyrrhizae Radix et Rhizoma, stir-fried Armeniacae Semen Amarum and gypsum (9, 6, 9 and 24 g); size-exclusion separation with light scattering and microscopy (Table 3)	Colloidal NPs; radius of gyration 50–150 nm	Association of ephedrine and pseudoephedrine and colloidal fraction	Cell-viability profiles compared with synthetic ephedrine	Most of the measured ephedrine (99.7%) and pseudoephedrine (95.5%) was associated with the particles. Fractionation and viability differences do not alone establish native equilibrium or assembly-dependent therapeutic efficacy	Zhou et al., 2014
Astragalus-Angelica drug pair	Decoction-derived; separate defined model	Codecoction-associated particles; Z-ligustilide-astragaloside IV model	Codecoction of Astragali Radix and Angelicae Sinensis Radix (5:1) for 10–120 min; ultrafiltration-retained particles; a separate 2:1 molecular model (Table 3)	Amorphous NPs	Decoction time, composition and size	Isoproterenol-induced myocardial fibrosis model	Supports a processing-material link. Retained amorphous character does not mean unchanged size; 2:1 describes the model, not the whole native fraction	Liang et al., 2023
Zexie Tang	Decoction-derived	Heat-associated fructan-triterpenoid assembly	Alismatis Rhizoma and Atractylodis Macrocephalae Rhizoma (5:2) decocted separately for 1 h, then mixed and heated at 100 °C for 30 min; reassembly experiments and molecular dynamics (Table 3)	Heated microparticles, approximately 1 μm	Processing and metabolite exposure	Oral bioavailability and mouse MASH outcomes	Supports functional particles and a proposed assembly mechanism. Reassembly alone does not establish necessity for the complete formula’s effect	Chen et al., 2026
Genkwa Flos ethyl acetate fraction with added GA	Semi-defined	Association involving GA and extract metabolites	Ethyl acetate fraction of an aqueous Genkwa Flos extract refluxed with GA in water for 1 h (Table 3)	NP-containing preparation	Composition and association	L-02 cell cytotoxicity	Greater cytotoxicity accompanied complexation; this does not establish clinical incompatibility of a complete two-drug preparation	Yang et al., 2021

Preparation labels describe the tested material: Engineered, formulated from defined molecules; Decoction-derived, isolated or characterized from a decoction; Extract-recombined, prepared by mixing previously extracted materials; Semi-defined, a specified metabolite combined with a complex extract. Hybrid and carbonized comparators contain additional carrier materials or newly generated chemical structures. A purified model and its corresponding extract are distinguished even when reported in the same study. The finding-and-limitation column separates an observation from an inference requiring further evidence; it is not a numerical risk-of-bias score. The composition and processing of the botanical drug preparations are given in Table 3.

**TABLE 2:** Interpretation of complementary measurements used to examine assemblies and aggregates in crude botanical drug preparations. Observations are condition-dependent and are not exclusive diagnostic criteria.

Measurement	Finite dispersed assemblies	Soluble molecular complexes	Kinetically trapped aggregates	Non-specific colloids	Macroscopic precipitates
Separation and recovery	A reproducible fraction and co-elution support association; separation can alter the state	Retention depends on molecular size, binding and the separation method	May be retained reproducibly; retention does not establish equilibrium	Recovery depends on size, filtration and centrifugal conditions	Sedimentation or retention depends on size, density and sampling time
Size distribution by light scattering or particle tracking	A narrow population is possible, but is not proof of an equilibrium assembly	May be below the method’s detection range; non-detection is not proof of absence	Can be narrow or broad; time and preparation dependence are informative	Broad or multiple populations may occur; neither is uniquely diagnostic	Large particles may distort scattering or exceed the measurement range
Microscopy	Reproducible shapes support structural description; drying artifacts remain possible	Individual complexes may not be resolved by routine particle imaging	Can have regular or irregular shapes; morphology alone does not distinguish them	Flocs or discrete particles may occur, with overlap between categories	Crystals or amorphous solids may be observed
Zeta potential	Interpret with medium, ionic strength, steric and hydration effects	A particle zeta-potential interpretation may be inappropriate	No unique magnitude distinguishesa trapped state	Low or high magnitudes are possible; values alone do not assigna state	Measurements requirea suitable dispersed fraction
Concentration dependence	An association threshold may occur; it does not alone establish equilibrium	Binding and dissociation depend on concentration and affinity	An apparent threshold may depend on mixing and observation time	Aggregation may be concentration-dependent and reproducible	Solubility, nucleation and processing history influence formation
Dilution and reconcentration	Reversible changes support dynamic association within the tested timescale	Dissociation depends on affinity, exchange rate and dilution range	Persistence, hysteresis or slow relaxation can occur	Redispersion and reaggregation depend on the medium and history	Dissolution or redispersion may occur after changing concentration or medium
Diffusion-ordered NMR	Common apparent diffusion can support association; exchange and signal overlap complicate interpretation	Bound and exchanging molecules can show shared or averaged diffusion	Slow diffusion or broadening is possible; no unique signature is expected	Broadening or signal loss can limit interpretation	The solid phase may be invisible to solution NMR
Reconstitution from defined molecules	Similar structures supporta proposed mechanism, not equilibrium or pharmacological necessity	Can reproduce molecular association under specified conditions	Can also be reproducible; reproduction alone does not exclude trapping	Reproducible colloids are possible without selective molecular recognition	Reproducible precipitation does not demonstratea dispersed assembly

These observations overlap across states. Interpretation therefore requires independent measurements, preparation metadata and time-dependent perturbations; no single entry establishes equilibrium or biological relevance.

TCM is considered here as a set of experimentally defined preparation contexts. For example, Ma-Xing-Shi-Gan-Tang, prepared from Ephedrae Herba, honey-processed Glycyrrhizae Radix et Rhizoma, stir-fried Armeniacae Semen Amarum and gypsum (Table 3), permits examination of how ephedrine and pseudoephedrine are distributed between soluble and colloidal fractions, the Astragalus-Angelica drug pair permits comparison across codecoction times, and Zexie Tang provides a model of heat-associated polysaccharide-triterpenoid assembly (Zhou et al., 2014; Liang et al., 2023; Chen et al., 2026). Their value lies in the particular combinations and preparation variables that can be tested. The underlying physical chemistry is also relevant to other botanical traditions and to food or pharmaceutical dispersions. We therefore discuss molecular interactions and architectures first, then assess physicochemical and biological comparisons, computational approaches, and the limitations that govern translation (Scheme 1).

**TABLE 3:** Composition and processing of the botanical drug preparations discussed in this review, as stated in the original studies and arranged according to the ConPhyMP reporting items (Heinrich et al., 2022).

Preparation (study)	Botanical drugs: accepted name [family; pharmaceutical name]	Source and identification as reported	Proportions and amounts	Processing	Material analyzed	Metabolites identified or quantified
Scutellariae Radix and Coptidis Rhizoma drug pair (Wei et al., 2025)	*Scutellaria baicalensis* Georgi [Lamiaceae; Scutellariae Radix]; *Coptis chinensis* Franch. [Ranunculaceae; Coptidis Rhizoma]; plant parts not stated	Purchased from Tong Ren Tang (Beijing, China); batch, authentication and voucher specimen not reported	Lyophilized single-drug decoctions combined 1:1 (w/w)	Each drug soaked in 8 volumes of water for 20 min and refluxed for 15 min; filtrates lyophilized. Powders redissolved in water and mixed immediately without heating, or preheated at 80 °C for 10 min, mixed and held at 80 °C for 30 min	Redissolved and mixed extracts; separate model of purchased baicalin and berberine hydrochloride (98%)	UHPLC-Q-Orbitrap HRMS profiling; baicalin and berberine contents compared by HPLC
Ma-Xing-Shi-Gan-Tang (Zhou et al., 2014)	*Ephedra sinica* Stapf [Ephedraceae; Ephedrae Herba], stem; *Glycyrrhiza uralensis* Fisch. ex DC. [Fabaceae; Glycyrrhizae Radix et Rhizoma Praeparata cum Melle], root and rhizome, honey-processed; *Prunus armeniaca* L. [Rosaceae; Armeniacae Semen Amarum], seed, stir-fried, reported as *P. armeniaca* var. Ansu Maxim.; gypsum (Gypsum Fibrosum, CaSO4·2H2O), a mineral	Purchased from Beijing Shuangqiao Yanjing Medicinal Material Factory; authenticated at Fujian University of Traditional Chinese Medicine; voucher specimens deposited at the university museum with identification codes	9 g, 6 g, 9 g and 24 g (one daily dose); water 1:8 (w/v)	Soaked for 30 min with stirring; Ephedrae Herba boiled for 30 min before the remaining materials were added; mixture boiled for a further 30 min, cooled and filtered through two layers of gauze	Decoction centrifuged at 400 *g* for 15 min and fractionated by size-exclusion chromatography with multi-angle light scattering (Sephacryl S-1000, citrate buffer, pH 5.0)	Ephedrine and pseudoephedrine measured by reversed-phase chromatography; 99.7% and 95.5%, respectively, associated with the isolated nanoparticles
Astragalus-Angelica drug pair (Liang et al., 2023)	*Astragalus mongholicus* Bunge [Fabaceae; Astragali Radix], reported as *Astragalus membranaceus* (Fisch.) Bge.; *Angelica sinensis* (Oliv.) Diels [Apiaceae; Angelicae Sinensis Radix]; plant parts not stated	Purchased from Baicaotang Chinese Herbal Medicine (Luzhou, China); batch numbers 221212–3 and 220,510–3; authentication and voucher specimen not reported	16.7 g and 3.3 g (5:1, the ratio of Danggui Buxue decoction) in 400 mL water	Soaked for 30 min, boiled for 10–120 min, centrifuged at 4,000 rpm for 10 min; supernatant ultrafiltered (MWCO 3500 Da) at 12,000 rpm for 40 min	Ultrafiltration-retained particles (AA-NPs)	43 metabolites identified by UHPLC-HR-MS with semiquantitative peak areas; Z-ligustilide and astragaloside IV used as a 2:1 molecular model
Zexie Tang (Chen et al., 2026)	*Alisma plantago-aquatica* L. [Alismataceae; Alismatis Rhizoma], dried rhizome; *Atractylodes macrocephala* Koidz. [Asteraceae; Atractylodis Macrocephalae Rhizoma], dried rhizome	Zhejiang Chinese Medical University Decoction Pieces Co., Ltd. (Hangzhou, China); batch numbers 240201 and 230,901; authentication and voucher specimen not reported	300 g and 120 g (5:2); water 1:10 (w/v)	Each drug decocted separately: soaked for 30 min and gently boiled (about 100 °C) for 1 h; filtered extracts mixed without further heating, or mixed and heated at 100 °C for 30 min	Heated microparticles collected at 1,600 × g for 30 min; nanoparticles at 120,000 × g for 30 min; particle-free supernatant as comparator	Alisol A, alisol B and 23-acetyl-alisol B identified in the particles; alisol A and alisol B quantified in plasma by LC-MS/MS; inulin-type fructan characterized by HPSEC, HPAEC and NMR
Genkwa Flos fraction with added glycyrrhizic acid (Yang et al., 2021)	*Daphne genkwa* Siebold & Zucc. [Thymelaeaceae; Genkwa Flos], plant part not stated; glycyrrhizic acid of >98% purity from a commercial supplier	Genkwa Flos purchased from Beijing Tong Ren Tang (Beijing, China); batch, authentication and voucher specimen not reported	25 mg ethyl acetate fraction and 20 mg glycyrrhizic acid in 10 mL water	50 g Genkwa Flos refluxed in 500 mL water for 1 h; filtrate concentrated to 50 mL and partitioned three times with 50 mL ethyl acetate; ethyl acetate fraction lyophilized, then refluxed with glycyrrhizic acid in water for 1 h	Glycyrrhizic acid and extract complex, compared with the fraction alone	58 metabolites identified by UHPLC-Q-Orbitrap HRMS; yuanhuacine and glycyrrhizic acid interaction examined by 1H NMR and NOESY

Accepted plant names follow the World Checklist of Vascular Plants; the name used in the original study is given where it differs. None of the five studies reported the legal basis for collection and processing of the plant material (ConPhyMP item 4).

**SCHEME 1:**
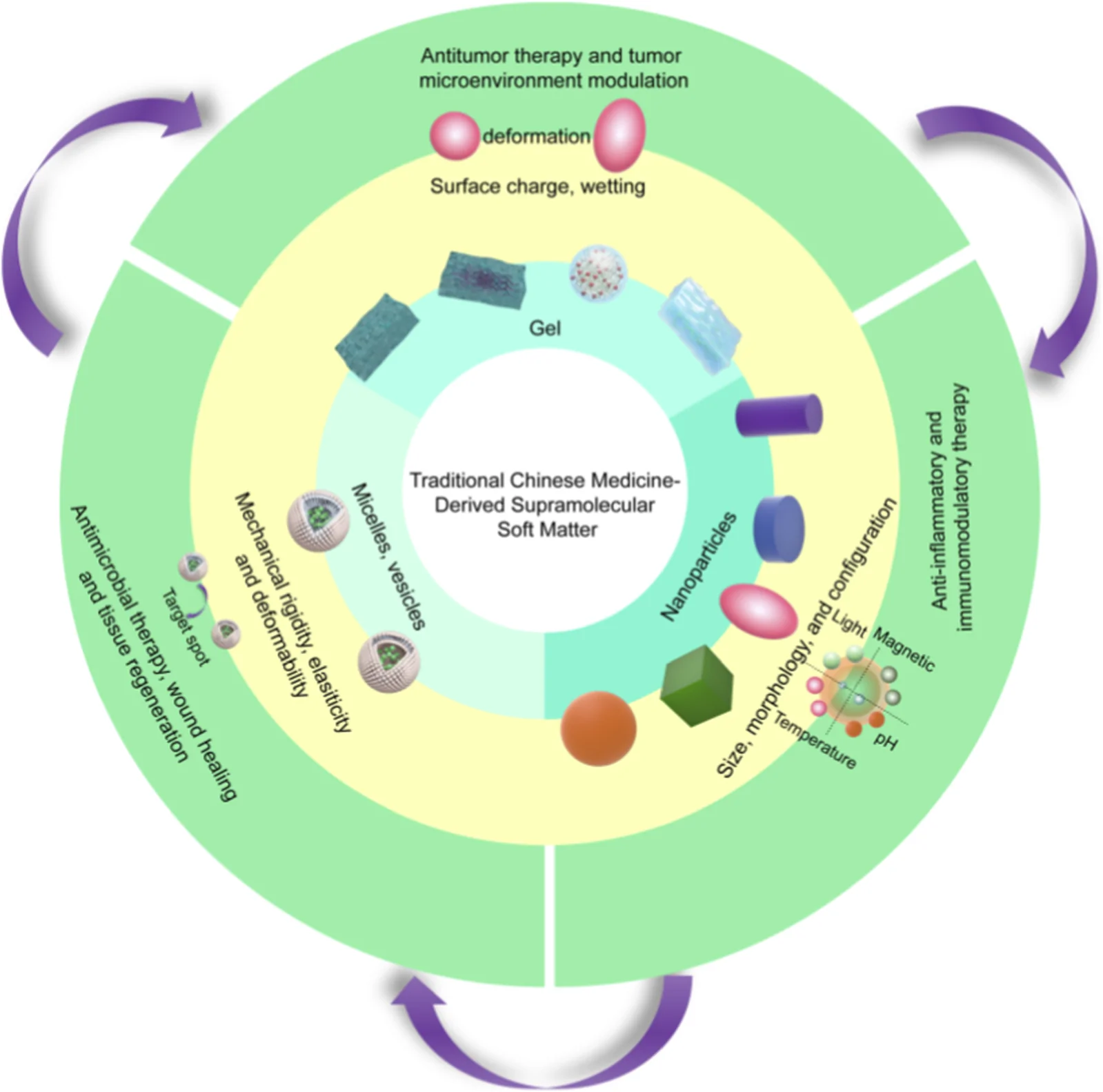
Overview of assembly architectures, physicochemical descriptors and biomedical applications discussed in this review. The scheme organizes topics for evaluation; its connections do not establish causal relationships shared by all preparations. TCM, traditional Chinese medicine; SSM, supramolecular soft matter.

Interactions among metabolites in botanical mixtures are well established and are not specific to TCM. They have been discussed mainly as pharmacodynamic synergy or antagonism, in which several metabolites act on the same or related targets, and as pharmacokinetic interactions, in which one metabolite alters the solubility, absorption or metabolism of another (Williamson, 2001; Wagner and Ulrich-Merzenich, 2009; Efferth and Koch, 2011; Yang et al., 2014; Caesar and Cech, 2019). These approaches treat a mixture as a set of co-present metabolites whose effects are analyzed as interactions between individually dissolved molecules. A supramolecular assembly is a different concept: it is a physically organized entity, formed by non-covalent association of metabolites, for example, during decoction, with its own size, morphology and surface properties, which can change the dose actually presented to cells or tissues. This physical level has received less systematic attention in the literature on mixtures. Colloidal aggregation is well documented in drug discovery, where it can cause nonspecific inhibition in screening assays and reduce the activity of dispersed drugs in cell culture (McGovern et al., 2002; Owen et al., 2012). This review does not claim that such assemblies are unique to TCM or that they replace pharmacological explanations of mixture effects. Its contribution is narrower: it examines where a physical association between metabolites of a botanical preparation has actually been measured, how such an association can be distinguished from pharmacodynamic and pharmacokinetic interactions, and what evidence would be needed to attribute part of a mixture effect to it.

This narrative review uses selected examples to examine how preparation source, structural measurements, and biological comparisons affect the interpretation of structure-function relationships. It is not an exhaustive systematic review and does not estimate how frequently particular assemblies occur. Primary studies inform the individual examples, while related reviews provide context. This approach permits comparison of experimental reasoning, but is vulnerable to selective coverage and cannot establish field-wide rates of assembly, clinical benefit, or methodological deficiencies. Botanical identity and terminology are retained at the level supported by the cited study.

Evidence is grouped by the material actually tested. Engineered or chemically defined assemblies are prepared from purified metabolites under controlled formulation conditions. Decoction-derived assemblies are isolated or characterized from a botanical drug decoction; the label describes provenance, not proof of equilibrium or pharmacological necessity. Extract-recombined preparations are made by mixing previously extracted materials, including redissolved freeze-dried extracts, and are distinguished from structures sampled directly from a codecoction. Semi-defined preparations combine a specified metabolite with a chemically complex extract. Engineered formulations containing a polymer, chemically modified metabolite, or carbonized material are additionally identified as hybrids or related comparators. A single paper may investigate more than one class, so evidence from a purified model is not assigned automatically to its corresponding extract. Table 1 applies these distinctions to the selected examples, and Box 1 defines the key terms used in this review.

Box 1 **Key terms and how they are used in this review.**

**Table:** 

Term	Meaning in this review
Botanical drug	A medicinal plant material, such as a dried root, rhizome, seed or flower, named by its pharmaceutical name together with the accepted species name, authority and family
Plant metabolite	A molecule produced by a plant, such as an alkaloid, flavonoid glycoside, saponin, terpenoid or polysaccharide
Mixture	Co-present metabolites considered as individual molecules; mixture effects such as synergy, antagonism and pharmacokinetic interactions are usually analyzed as interactions between dissolved molecules
Supramolecular assembly	An organized structure formed by two or more molecules held together mainly by non-covalent interactions, such as hydrogen bonding, hydrophobic, electrostatic and π-π interactions
Supramolecular soft matter (SSM)	Assemblies whose structure and properties, such as size, morphology and deformability, arise from weak, reversible interactions and change with concentration, temperature and medium
TCM-derived SSM	SSM containing plant metabolites from botanical drugs used in TCM; the term describes the origin of the metabolites, not a property unique to TCM.
Engineered formulation	A formulation prepared from purified metabolites under controlled conditions
Decoction-derived assembly	An assembly isolated or characterized directly from a decoction
Extract-recombined preparation	A preparation made by mixing previously extracted and redissolved materials
Semi-defined preparation	A specified metabolite combined with a chemically complex extract
Hybrid or carbonized comparator	A material containing a synthetic carrier or a chemically transformed product; it is not a reversible assembly of unmodified metabolites
Carrier-free	Without a separate carrier matrix
Compatibility	The traditional principle of combining botanical drugs in a formula (peiwu)

## 2 Molecular basis and supramolecular driving forces in TCM-Derived SSM

### 2.1 Amphiphilic and super-amphiphilic plant metabolites as self-assembling building blocks

Amphiphilicity can favor the spontaneous organization of bioactive plant metabolites into TCM-derived SSM. Molecules with spatially separated hydrophilic and hydrophobic domains can reduce unfavorable water exposure through hydrophobic association while maintaining colloidal dispersion through hydrophilic groups. This hydrophilic–hydrophobic balance influences interfacial curvature, packing density, critical aggregation behavior, and the formation of micelles, vesicles, nanoparticles, or other nanoassemblies (Wang et al., 2009; Guo et al., 2024; Wang Y.-L. et al., 2025). The resulting morphology also depends on concentration, ionization, molecular geometry, solvent, and preparation history; amphiphilicity alone does not specify which structure will form.

In addition to intrinsically amphiphilic molecules, super-amphiphilic building blocks can be generated through non-covalent complexation between hydrophilic and hydrophobic motifs. This process can enable reversible modulation of amphiphilicity and assembly–disassembly behavior (Zhang and Wang, 2011). In TCM-derived SSM, saponins, glycosides, terpenoids, and alkaloid–polyphenol pairs can either contain inherent amphiphilic domains or acquire amphiphile-like characteristics through intermolecular association. Effective amphiphilicity therefore depends on molecular composition, metabolite ratio, glycosylation pattern, ionization state, and environmental conditions. Reversibility must be demonstrated under the conditions studied.

Saponins provide representative examples of amphiphilic TCM-derived building blocks. Glycyrrhizic acid (GA), a bioactive saponin associated with Glycyrrhizae Radix et Rhizoma, contains a hydrophobic triterpenoid aglycone and hydrophilic glucuronic acid residues (Ni et al., 2022). This amphiphilic architecture provides the structural basis for micellar self-assembly and drug solubilization (You et al., 2021). More broadly, saponins possess natural block-copolymer-like architectures in which the aglycone acts as the hydrophobic segment and sugar chains act as hydrophilic segments (Böttcher and Drusch, 2017). Ginsenoside Rb1 has likewise been investigated in an engineered micellar delivery formulation (Li M. et al., 2019). These examples make the balance between sugar moieties and the aglycone a useful formulation variable; results obtained with purified saponins do not establish the concentration, persistence, or biological role of the same structures in a complete decoction.

### 2.2 Non-covalent interactions and dynamic bonding networks

Electrostatic attraction, hydrogen bonding, hydrophobic association, and π-π interactions can contribute jointly to the assembly of plant metabolites (Gao et al., 2022; Dong et al., 2024; Zhang et al., 2024c). Metal coordination and reversible covalent linkages provide additional mechanisms in engineered formulations, but should be identified separately. Assigning a driving force requires comparison of measurements and perturbations; a change in a spectroscopic band alone does not establish a unique assembly mechanism or biological consequence.

Electrostatic interactions are particularly important for co-assembly between oppositely charged plant metabolites. Positively charged alkaloids, such as berberine (BBR), can interact with negatively charged carboxylate- or phenolate-containing molecules, thereby promoting charge compensation and molecular association (Chen S. et al., 2022; Dong et al., 2024). Because the protonation states of carboxyl, phenolic hydroxyl, and amino groups are pH-dependent, electrostatic interactions can also contribute to pH-regulated assembly and disassembly behavior. Charge complementarity may promote a dispersed assembly or precipitation, depending on stoichiometry, concentration, ionic strength, and the other interactions present. It therefore cannot, on its own, predict a nanoparticle rather than a fibrous network or hydrogel.

Hydrogen bonding and π–π stacking can stabilize supramolecular packing. Hydrogen bonds formed among hydroxyl, carbonyl, carboxyl, glycosidic, and phenolic groups can guide the directional organization of polysaccharides, glycosides, flavonoids, terpenoids, and polyphenols (Shahidi and Athiyappan, 2025; Zhu et al., 2026). Multiple hydrogen-bonding motifs can contribute to hydrogelation, nanofiber formation, and capsule-like structures (Liu Y. et al., 2024). In parallel, π–π stacking between aromatic rings in alkaloids, flavonoids, phenolic acids, and tannins can promote close molecular packing. These interactions can cooperate with hydrophobic association and electrostatic attraction (Chen S. et al., 2022; Zhang et al., 2024c). Their presence does not by itself demonstrate ordered growth, reversibility, or stability, all of which require measurements of the resulting material under stated conditions.

Dynamic coordination and reversible covalent networks further expand the structural diversity of engineered TCM-derived materials. Metal–phenolic networks (MPNs) are formed through coordination between metal ions and phenolic ligands, providing a strategy for constructing coatings, capsules, NPs, and hydrogels (Geng et al., 2022). Polyphenols such as tannic acid, curcumin, and catechins can participate in these networks, with formulation-dependent redox or pH responses (Kim et al., 2015; Shen et al., 2022). Boronate–phenolic networks (BPNs), formed between boronic acid groups and suitable diols or catechol-containing polyphenols, represent another dynamic bonding motif. Boronate ester bonds are reversible covalent linkages and should not be described as purely non-covalent self-assembly (Guo et al., 2015). Both approaches are relevant delivery strategies, but their introduced reagents and reaction conditions must be distinguished from those of unmodified botanical drug decoctions.

Mechanistic interpretation is strongest when a proposed interaction is perturbed and the resulting changes in structure and function are measured together. Solvent, pH, salt, temperature, and mixing changes can affect several interactions simultaneously, so these experiments also require controls for solubility and chemical composition. The interaction categories above provide hypotheses for testing, rather than a universal explanation for the morphology or activity of TCM preparations.

### 2.3 Molecular recognition, carrier minimization, and stereochemical control

Molecular recognition can translate general non-covalent interactions into selective and directional assembly pathways. In defined TCM-derived formulations, recognition arises from the spatial matching of charges, aromatic surfaces, hydrogen-bonding sites, hydrophilic–hydrophobic domains, and stereochemical configurations. This selective matching can influence assembly stoichiometry and molecular packing, enabling bioactive plant metabolites to act as both therapeutic molecules and structural building blocks (Zhang et al., 2024c; Wang Y.-L. et al., 2025). Carrier minimization is a separate formulation property: it depends on the amount and function of every material in the formulation, not simply on whether a plant metabolite is present. A high metabolite fraction does not itself prove selective targeting, improved safety, or a structure-dependent therapeutic effect.

In carrier-minimized formulations, selected alkaloids, polyphenols, saponins, polysaccharides, and flavonoids can participate directly in supramolecular construction through complementary recognition motifs (Zhang et al., 2024c; Zhu et al., 2026). Representative examples include self-assembled GA–aconitine assemblies, botanical-drug-pair-inspired supramolecular prodrugs, and botanical-drug-derived NPs from *Scutellaria barbata* D. Don [Lamiaceae; Scutellariae Barbatae Herba] and *Scleromitrion diffusum* (Willd.) R.J.Wang [Rubiaceae; Hedyotidis Diffusae Herba] (Qian et al., 2025; Xia et al., 2025; Liu et al., 2026). These formulations differ in preparation and chemical modification. Their performance should be attributed to the tested material rather than treated as a shared consequence of molecular recognition.

Stereochemistry provides a useful controlled variable. Lu et al. (2024) compared the assembly behavior of 1,2,3,4,6-penta-O-galloyl-β-D-glucose and its α-D-glucose epimer. Their different gelation behavior supports a relationship between molecular configuration and network formation in that engineered gel. This comparison is more specific than simply observing that a plant extract gels. Nevertheless, a configuration-dependent material change should be distinguished from proof that gelation, rather than another effect of the epimer, accounts for a biological endpoint.

Overall, molecular recognition, carrier minimization, and stereochemical control should be considered interconnected design principles. Molecular recognition concerns selective association of metabolites; carrier minimization concerns the amount and function of added formulation materials; and stereochemical control concerns how molecular geometry affects packing. Together, these principles bridge the molecular driving forces discussed above with the hierarchical architectures summarized in the following section. Extending a mechanism from a defined formulation to a preparation requires evidence that the relevant molecules, concentrations, and physical states occur there.

### 2.4 Endogenous supramolecular organizers and the supramolecular reading of botanical drug compatibility

The principles described above raise a question for multicomponent traditional preparations: whether certain metabolites act not only as pharmacologically active molecules but also as endogenous supramolecular organizers, that is, as bioactive excipients that alter the physical state, solubility, stability, transport and exposure of other metabolites in the same formula. GA is one candidate because it combines a hydrophobic triterpenoid aglycone with hydrophilic glucuronic acid residues (Section 2.1). A supramolecular reading of compatibility therefore asks which metabolites are pharmacologically active and which may organize others. This is a testable physical interpretation of compatibility, not a general explanation of every traditional drug combination.

In Zexie Tang, thermal decoction was reported to generate particles involving an inulin-type fructan from *Atractylodes macrocephala* Koidz. [Asteraceae; Atractylodis Macrocephalae Rhizoma] and alisol A, alisol B and 23-acetyl-alisol B from *Alisma plantago-aquatica* L [Alismataceae; Alismatis Rhizoma]. The heated microparticles, approximately 1 μm in size, were associated with improved oral triterpenoid bioavailability and activity in a mouse model of metabolic dysfunction-associated steatohepatitis (MASH). Reassembly experiments and molecular dynamics supported proposed interactions involved in particle formation (Chen et al., 2026). These findings connect preparation, composition, and preclinical activity. They should not be converted into a claim that the assemblies are the sole or necessary cause of the full formula’s efficacy; that conclusion requires an independently evaluated comparison that selectively changes assembly while controlling chemical exposure.

Other studies address different parts of this question. Nanoparticles isolated from a codecoction of *Astragalus mongholicus* Bunge [Fabaceae; Astragali Radix], reported as *Astragalus membranaceus*, and *Angelica sinensis* (Oliv.) Diels [Apiaceae; Angelicae Sinensis Radix] changed with decoction time and were evaluated in myocardial fibrosis models; the reported 2:1 Z-ligustilide-astragaloside IV complex was a defined mechanistic model, not the overall stoichiometry of the native fraction (Liang et al., 2023). A purified BBR-aristolochic acid model reduced acute nephrotoxicity in experimental models, supporting investigation of molecular association without establishing the safety of aristolochic-acid-containing preparations (Wang et al., 2021). Refluxing GA with the ethyl acetate fraction of an aqueous extract of *Daphne genkwa* Siebold & Zucc. [Thymelaeaceae; Genkwa Flos] produced a semi-defined preparation with increased L-02 cell toxicity (Yang et al., 2021). These observations concern different materials and endpoints and cannot be combined as if they constituted one complete formula-level causal experiment.

A causal comparison would change the assembled state while controlling metabolite identity, dose and exposure, then determine whether biological activity changes and can be restored. Depletion and add-back studies are informative only if compositional and processing effects of the manipulation are controlled. The examples above support investigation of compatibility-related physical states. Evidence of association, mechanistic support from a defined model, and demonstration of necessity in a whole preparation nevertheless remain distinct levels of inference.

## 3 Hierarchical architectures of TCM-derived supramolecular soft matter

### 3.1 Micellar and vesicular compartmentalization

Micellar and vesicular compartmentalization represents a hierarchical architecture formed by amphiphilic bioactive plant metabolites, including selected saponins. Above an experimentally determined critical micelle concentration (CMC), suitable saponins can self-assemble through hydrophobic association into micellar structures composed of a hydrophobic inner core and a hydrophilic outer shell. This compartmentalized architecture enables hydrophobic molecules to be accommodated within the core while maintaining colloidal dispersion in aqueous environments, thereby supporting solubilization and formulation compatibility (Böttcher and Drusch, 2017). The extent of solubilization and persistence of the dispersion depend on the saponin, concentration and medium.

Representative saponin-based formulations illustrate how molecular structure can influence micellar behavior. The formulations in this subsection were prepared from purified metabolites under controlled conditions rather than sampled from a decoction. Quillaja saponin-based delivery systems can encapsulate lutein esters, demonstrating the ability of natural saponins to solubilize hydrophobic molecules (Tippel et al., 2016). Ginsenoside-based self-assembled NPs have also been studied for insulin delivery (Dai et al., 2016; Zou et al., 2021). Similarly, GA can form micellar assemblies for loading hydrophobic drugs such as baicalein (You et al., 2021), while saponin-based micelles more broadly serve as delivery compartments for hydrophobic bioactive molecules (Liao et al., 2021). These studies support specific solubilization and delivery applications. The amount and function of every added material must be considered before describing a formulation as carrier-free.

Beyond spherical micelles, saponins can form vesicular or multicompartment architectures. Dissipative particle dynamics (DPD) simulations of platycodin (saponins from *Platycodon grandiflorus* (Jacq.) A. DC [Campanulaceae; Platycodonis Radix]) assemblies have shown that morphology is strongly influenced by the hydrophilic-to-hydrophobic ratio of the saponin structure and the drug-to-saponin ratio during formulation (Dai et al., 2015). A higher proportion of hydrophilic sugar moieties tends to favor vesicles with more internal compartments, whereas changes in drug-to-saponin ratio can alter the diameter and packing pattern of the resulting assemblies. Thus, micelle-to-vesicle transitions are governed by the balance among molecular amphiphilicity, interfacial curvature, and formulation stoichiometry.

Overall, micellar and vesicular compartmentalization provides a structural mode through which amphiphilic plant metabolites organize bioactive molecules within nanoscale domains. These architectures provide a basis for discussing the broader hierarchy of TCM-derived SSM, including the solid NPs and NFs discussed in the next section (Figure 1c). The examples above do not establish that the same structures persist at effective concentrations in an authentic decoction or after administration. Matched free-metabolite and disrupted-assembly comparisons are needed before attributing efficacy specifically to micellar or vesicular organization.

**FIGURE 1:**
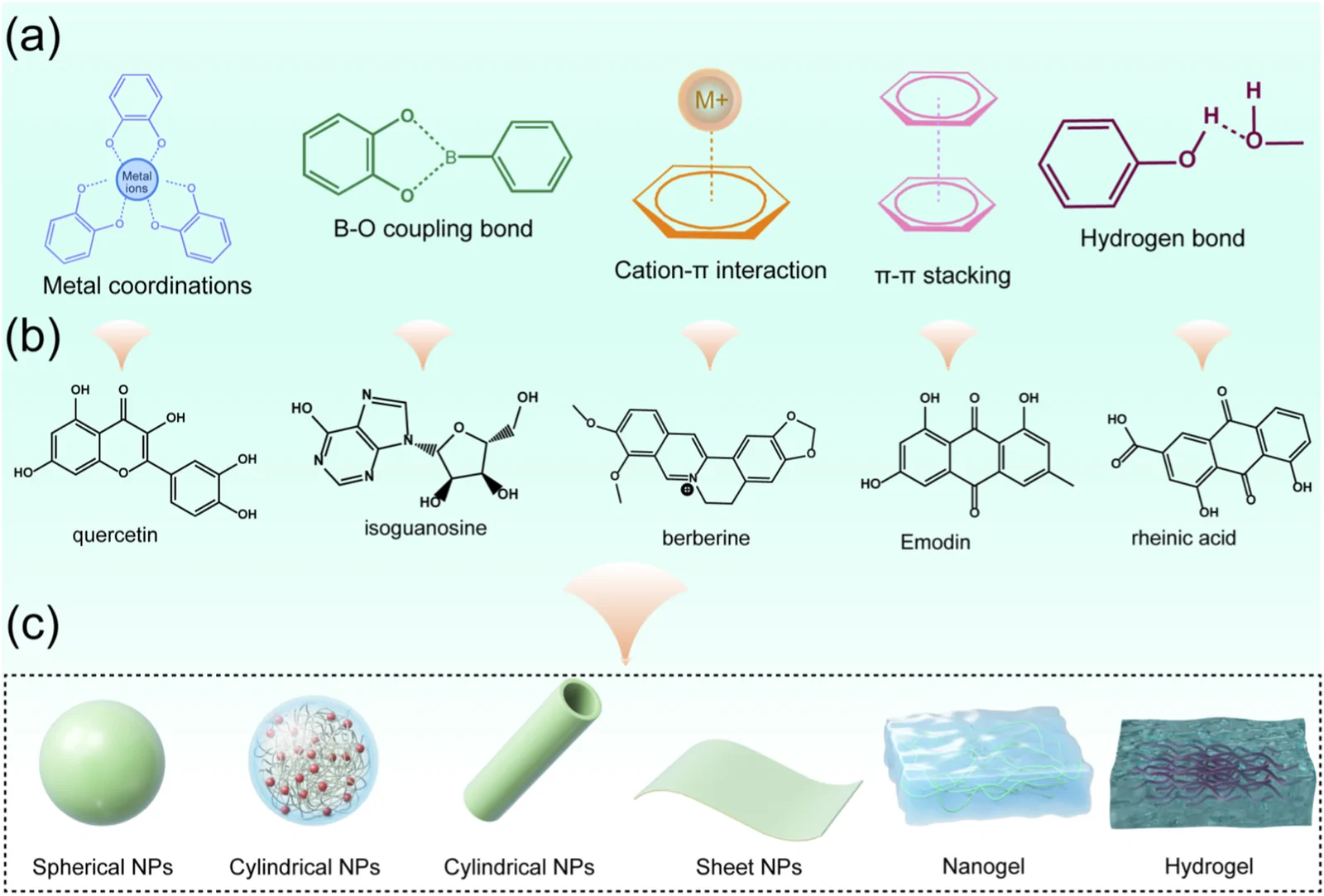
Schematic illustration of plant-metabolite assembly and related bonding mechanisms. **(a)** Non-covalent interactions, metal coordination and boronate ester bonding; boronate esters are reversible covalent linkages. **(b)** Molecular building blocks. **(c)** Possible architectures. The structures shown are examples rather than a claim that every metabolite forms each architecture.

### 3.2 Solid nanoparticles, nanofibers, and carbon-based nanoassemblies

Beyond micellar and vesicular compartments, bioactive plant metabolites can also organize into solid NPs and NFs. Their formation can involve cooperative hydrophobic association, π–π stacking, hydrogen bonding, electrostatic attraction, and coordination interactions. Depending on molecular geometry and assembly conditions, bioactive plant metabolites can generate spherical NPs, rod-like assemblies, or flower-like hierarchical structures through direct self-assembly or co-assembly with other metabolites (Bag and Dash, 2015; Wang et al., 2020; Cheng et al., 2021). These morphologies do not establish a fixed hierarchy of colloidal stability or therapeutic performance. Size distribution, concentration, preparation history, and chemical composition must accompany morphological measurements if biological comparisons are to be interpreted.

Wei et al. (2025) compared unheated and heated mixtures prepared from redissolved freeze-dried aqueous extracts of *Scutellaria baicalensis* Georgi [Lamiaceae; Scutellariae Radix] and *Coptis chinensis* Franch [Ranunculaceae; Coptidis Rhizoma], mixed 1:1. This extract-recombined experiment was accompanied by a separate purified baicalin-BBR model. Heating was associated with a shift from fibrous to spherical structures, and the antibacterial preference differed between *Staphylococcus aureus* and *Bacillus subtilis*. Thus, the study supports a preparation-dependent morphology comparison, not a general claim that spherical particles are superior or that the measured structures were sampled directly from a freshly prepared codecoction.

Carbon-based nanoassemblies constitute a distinct class of TCM-derived nanostructures generated during high-temperature processing or carbonization of botanical drugs. These structures are often referred to as TCM-derived carbon dots or carbon quantum dots and usually possess ultrasmall particle sizes, abundant surface functional groups, and tunable optical or redox properties. Unlike purely non-covalent assemblies, carbon-based nanoassemblies are processing-induced carbonaceous nanostructures; nevertheless, their size, surface chemistry, and aggregation state can influence biological performance (Liu et al., 2018; Mishra et al., 2018; Zhang et al., 2019). These carbonized particles are adjacent to, rather than examples of, reversible plant-metabolite self-assembly: high-temperature processing produces new carbon domains whose identity is no longer that of the parent metabolite. They are retained here only to mark the boundary of the TCM-derived SSM definition.

Particle morphology alone does not distinguish reversible assembly from a kinetically trapped aggregate or processing-generated material. For carbonized products, the new chemical identity must also be characterized. Comparisons should therefore specify what material was prepared and which structure was measured, and avoid treating a shared nanoscale size as proof of a common formation mechanism or biological action.

### 3.3 Hydrogels, nanogels, oleogels, and organogels

Supramolecular gel networks connect molecular assembly with macroscopic material behavior. Unlike micelles, vesicles, or discrete NPs, these gels can form through the percolation of self-assembled fibrils, tubular aggregates, vesicular clusters, or crosslinked nanoassemblies into three-dimensional networks. Depending on the continuous phase and network scale, they can be classified into hydrogels, nanogels, oleogels, and organogels (Figure 1c). Water or solvent retention, injectability, self-healing behavior, and local retention are potentially useful formulation properties, but each requires measurement. In polymer-containing or chemically crosslinked gels, the network contribution of the carrier must be distinguished from self-assembly of the plant metabolite.

The gel examples below are engineered formulations rather than gels directly isolated from decoctions. Amygdalin and GA have been formulated as a self-assembled hydrogel, whereas chitosan-puerarin gels contain a separate macromolecular partner (Luo et al., 2026; Chen B. et al., 2022). Zingibroside R1 (ZR1) provides an example of a metabolite-derived nanofibril whose structure was resolved by cryo-electron microscopy (Peng et al., 2025). These studies differ in composition and structural resolution and should not be grouped under carrier-free delivery without checking the role of all formulation materials.

Nanogels represent nanoscale gel networks that combine the structural softness of gels with the transport behavior of nanoassemblies. Oleanolic acid (OA) nanoassemblies prepared from the purified metabolite, including relatively rigid OA nanoparticles (OA-NPs) and flexible OA nanogels (OA-NGs), provide an example of mechanically different architectures made from the same metabolite. Their comparison links measured mechanical behavior to cellular transport and antitumor activity, as discussed in Section 4.3 (Li et al., 2021). It does not establish that all nanoscale gels share those biological properties.

Oleogels and organogels provide structurally relevant natural-product gel models for extending TCM-derived SSM into non-aqueous or oil-rich environments. Phytosterol-based oleogels can be formed through hydrogen bonding and hydrophobic packing among sterol molecules, generating tubular or fibrous networks that immobilize the oil phase and regulate mechanical properties (Yang et al., 2018). Organogels are typically generated by low-molecular-weight terpenoid or steroidal gelators in organic solvents through hydrogen bonding, van der Waals interactions, and hydrophobic association (Bag et al., 2009). These interactions can drive the formation of three-dimensional fibrous networks or vesicle-based gel structures, thereby informing the design of TCM-derived supramolecular gels with broader phase environments and mechanical profiles.

Overall, supramolecular gels bridge nanoscale assembly and macroscopic material behavior. Hydrogels, nanogels, oleogels, and organogels provide measurable descriptors such as gelation behavior, network density, viscoelasticity, swelling capacity, injectability, and local retention. These measurements characterize network-level properties; a gel’s retention or biological response cannot automatically be attributed to non-covalent metabolite assembly. Polymer content, crosslinking, solvent residues, drug release, and dose are possible contributors. Comparison with the free metabolite, the carrier alone where applicable, and a suitable non-gelling formulation helps define which claim a gel study supports. Non-aqueous organogel behavior also does not establish persistence in physiological media.

### 3.4 Distinguishing genuine supramolecular species from non-specific aggregates in crude preparations

The architectures described above are defined by how molecules are organized, not by how strongly they interact. A strong intermolecular interaction is therefore not sufficient evidence that a stable nanoscale assembly has formed. Self-assembly is governed by a thermodynamic and kinetic landscape in which the same pair of metabolites may give rise to a stable finite-sized co-assembly, a soluble molecular complex, a kinetically trapped aggregate, non-specific colloidal aggregation, or macroscopic precipitation, depending on concentration, temperature, ionic strength, and the order and duration of mixing. The distinction becomes critical when the sample is not a two-component model but a crude extract or a decoction, in which large numbers of metabolites, polysaccharides and proteins coexist, and in which visible turbidity, a light-scattering signal, or a shifted spectroscopic band may each arise from several of these states simultaneously.

Compositional analysis often uses separation of particle-associated material from the free-solute background. For example, size-exclusion chromatography with multi-angle light scattering isolated colloidal fractions from Ma-Xing-Shi-Gan-Tang, with much of the measured ephedrine and pseudoephedrine associated with those fractions (Zhou et al., 2014). Chromatography-mass spectrometry and nuclear magnetic resonance can then characterize the recovered material, as illustrated by the GA-Genkwa Flos preparation (Yang et al., 2021). Separation is not a neutral step: dialysis, dilution, filtration, centrifugation, or chromatography may change association equilibria. Measurements in the original preparation and after separation, mass recovery, and analysis of the free fraction are therefore needed to assess how representative an isolated fraction is.

State assignment requires converging measurements and controlled perturbations. Light scattering measures an intensity-weighted size distribution and may be strongly influenced by larger particles; microscopy provides morphology but can introduce drying or sampling effects. Neither is sufficient alone to identify molecular composition or an equilibrium state. Zeta potential must be interpreted with the medium, steric effects, and hydration, while concentration-dependent association does not automatically establish thermodynamic equilibrium. Dilution and reconcentration, time-resolved measurements, and changes in temperature or ionic strength test different aspects of persistence and reversibility. Reconstitution from purified molecules can support a proposed assembly mechanism, but even a reproducible structure can be kinetically trapped. Table 2 therefore presents possible observations and limitations, rather than a diagnostic checklist with mutually exclusive outcomes.

The selected decoction studies illustrate different evidence levels. Zhou et al. (2014) demonstrated isolated colloidal fractions and the association of ephedrine and pseudoephedrine with them; this observation alone does not establish equilibrium. Liang et al. (2023) reported similar amorphous features across decoction times alongside possible size-distribution differences, so retained amorphous character should not be equated with unchanged particle size. Chen et al. (2026) reported functional heat-associated particles and mechanistic reassembly evidence; a general stability claim still requires specification of medium, concentration, temperature, and observation time. We distinguish detection of an assembly, persistence over a measured interval, reversible response to perturbation, and evidence consistent with equilibrium. These are related but non-equivalent conclusions.

The analytical limitation is that a crude preparation may contain several interconverting populations and no single readout assigns all of them uniquely. Processing history must also be specified: findings from aqueous decoction do not automatically apply to carbonization or to other paozhi (traditional processing) procedures involving different auxiliary materials or temperatures. This review therefore evaluates the particular preparation studied and avoids assigning stability or a mechanism to an entire processing category without condition-specific measurements.

## 4 Physicochemical determinants of biological fate

### 4.1 Size, morphology, and spatial configuration

Size, morphology, and spatial configuration are primary physicochemical descriptors that can influence how TCM-derived SSM interact with biological systems. Particle size can influence colloidal stability, tissue distribution, cellular uptake, and clearance, whereas morphology and spatial configuration can affect surface presentation, multivalent contact, membrane adhesion, and biological recognition (Wang C. et al., 2025). In TCM-derived assemblies, these descriptors are often interdependent: changes in molecular packing can simultaneously alter particle dimension, exposed functional groups, and interfacial curvature. A formulation-level difference therefore does not establish an independent effect of shape unless other influential variables are controlled.

Two BBR studies illustrate why the experimental source matters. Li T. et al. (2019) studied defined BBR-flavonoid glycoside assemblies and related their different antibacterial behavior to molecular organization and surface presentation. Wei et al. (2025) instead included recombined botanical extracts as well as a purified baicalin-BBR model. Its heated and unheated samples showed different antibacterial preferences across the tested species. The mechanisms and outcomes should be attributed to their respective preparations, rather than combining one paper’s extract protocol with another paper’s molecular explanation under a single native-assembly label.

These comparisons motivate study of morphology as a candidate functional variable. They do not demonstrate a universal advantage of NPs over NFs or establish a quality specification for all botanical preparations. Matched composition, dose, size distribution, surface chemistry, and species-specific assays are needed to identify what a morphology comparison actually explains (Figure 2).

**FIGURE 2:**
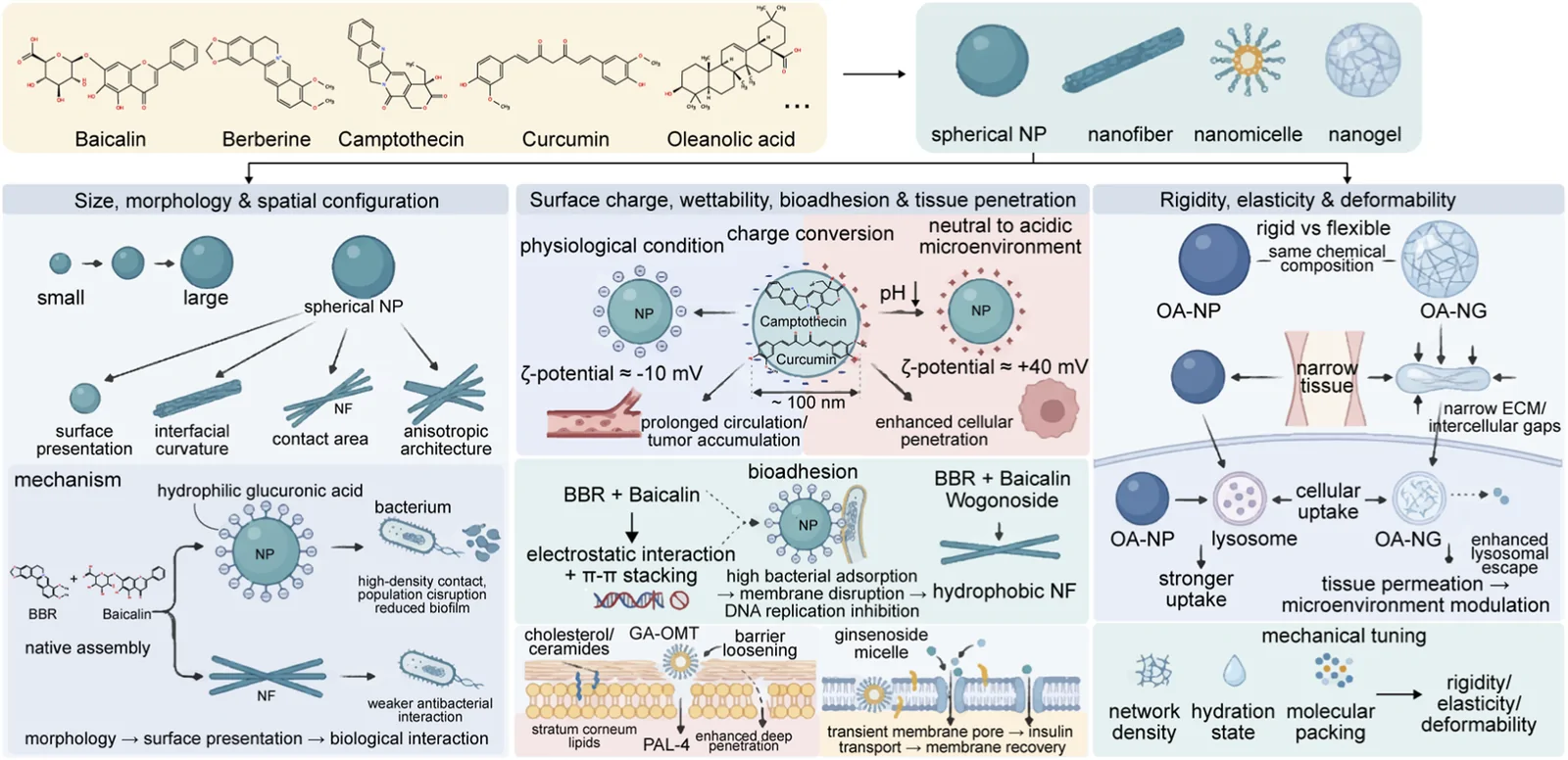
Physicochemical properties of plant-metabolite supramolecular assemblies. Left: assembly size, morphology and spatial configuration, comparing BBR-flavonoid glycoside nanoparticles and nanofibers prepared from defined molecules (Li T. et al., 2019) and baicalin-BBR assemblies from recombined extracts (Wei et al., 2025); in the latter study, the antibacterial advantage of each morphology depended on the bacterial species tested. Middle: surface charge, wettability, bioadhesion and tissue penetration, illustrated by charge-conversional curcumin NPs containing irinotecan, a camptothecin derivative (Xiao et al., 2020), GAO nanomicelles for transdermal peptide delivery (Wang Z. et al., 2025) and ginsenoside micelles for insulin transport (Zou et al., 2021). Right: rigidity, elasticity and deformability, comparing rigid OA-NPs with flexible OA-NGs (Li et al., 2021). Each part summarizes a specific study under its reported conditions. The arrows indicate relationships proposed in those studies and do not establish properties shared by all decoctions. BBR, berberine; ECM, extracellular matrix; GAO, glycyrrhizic acid-oxymatrine ionic liquid; NF, nanofiber; NP, nanoparticle; OA-NG, oleanolic acid nanogel; OA-NP, oleanolic acid nanoparticle; PAL-4, palmitoyl pentapeptide-4.

### 4.2 Surface charge, wettability, bioadhesion, and tissue penetration

Surface charge, wettability, and bioadhesion are interfacial descriptors that can influence how TCM-derived SSM interact with biological barriers. Surface charge can affect colloidal stability, protein adsorption, biodistribution, and cellular internalization, whereas wettability and bioadhesion can influence contact with membranes, bacteria, skin barriers, and diseased tissues. These descriptors are relevant to self-delivering or carrier-minimized formulations because bioactive plant metabolites can function as both therapeutic molecules and surface-presenting building blocks. Their effects depend on the surrounding medium and may change after adsorption of proteins or other biomolecules. A surface measurement in water is not, by itself, evidence of targeting or penetration *in vivo* (Figure 2).

Xiao et al. (2020) studied defined camptothecin-derivative/curcuminoid formulations, including irinotecan-curcumin NPs. The surfactant-stabilized preparation contained poloxamer 105, and its reported size and charge response should not be assigned indiscriminately to surfactant-free particles. The authors related pH-dependent charge behavior to transport and treatment outcomes in mice. This example supports investigation of responsive interfaces, while emphasizing that the stabilizer, medium and formulation composition are part of the tested formulation; it is not evidence that unmodified botanical preparations have the same targeting behavior.

In the chemically defined assemblies studied by Li T. et al. (2019), BBR-flavonoid glycoside NPs and NFs had different antibacterial behavior. The authors associated the NP surface presentation of hydrophilic glucuronic acid groups with stronger bacterial affinity. This is a formulation-specific interpretation, not evidence that every BBR-containing decoction has the same surface architecture. It should also be distinguished from the extract-recombination and heating comparison reported by Wei et al. (2025).

Surface interactions also control tissue penetration across biological barriers. Insulin-loaded ginsenoside micelles can bind membrane cholesterol and transiently form membrane pores, thereby facilitating insulin transport while allowing membrane integrity to recover (Zou et al., 2021). Similarly, GA and oxymatrine (OMT) form a GA–oxymatrine bioactive ionic liquid (GAO), which further self-organizes into GAO nanomicelles. These nanomicelles weakly interact with stratum corneum lipids, including cholesterol and ceramides, loosening the skin barrier and creating a nanoscale “pulling” effect that enhances the deep penetration of signal peptides such as palmitoyl pentapeptide-4 (PAL-4) (Wang Z. et al., 2025).

These studies support measurement of interfacial properties alongside delivery outcomes. The limitation is that charge conversion, hydration, metabolite release, and composition may change together. To establish an interfacial descriptor as a useful quality attribute, its measurement must be reproducible in a defined medium and linked to a relevant functional endpoint across independently prepared batches. A favorable result in one model is insufficient to set a general acceptance limit.

### 4.3 Mechanical rigidity, elasticity, and deformability

Mechanical rigidity, elasticity, and deformability are physicochemical descriptors relevant to the biological fate of TCM-derived SSM. Deformable supramolecular assemblies can undergo shape adaptation under external stress, potentially changing interfacial contact, transport through confined biological spaces, and interactions with cells or tissues. These mechanical behaviors are closely related to network density, hydration state, molecular packing, and interfacial adhesion, and therefore provide an additional structure–function dimension beyond size, morphology, and surface chemistry (Figure 2) (Deshmukh et al., 2015). Mechanical measurements need stated conditions and should be interpreted with other physicochemical descriptors rather than as an intrinsic advantage shared by all TCM-derived materials.

Deformability has been recognized as an important variable in nanomedicine delivery. Flexible nanodroplets or emulsion-like formulations can adhere to cells, deform under compression, and pass through narrow endothelial or intercellular gaps, thereby improving lymph-node delivery and immune activation (Xia et al., 2018; Song et al., 2021; Wu et al., 2025). Although not all of these formulations contain plant metabolites used in TCM, they provide a useful mechanistic basis for understanding why deformability may be important for TCM-derived SSM.

Li et al. (2021) compared relatively rigid OA-NPs with flexible OA-NGs. The nanogels showed less cellular uptake but greater lysosomal escape, tumor penetration and antitumor activity. This result shows why uptake alone is an incomplete performance measure. Although both formulations used OA, their preparation also differed; packing, hydration and other correlated attributes need consideration when assigning the biological difference specifically to mechanics.

The principal limitation is transferability. A mechanics-function relationship measured in engineered OA formulations requires separate testing in multicomponent preparations and in relevant biological media. Mechanical descriptors are consequently candidate measurements, not yet universal critical quality attributes. Independent batches, matched-composition controls, measurements after biological exposure, and tests of whether mechanics adds predictive value beyond size or composition would strengthen the inference (Figure 2; Table 1).

### 4.4 Stimuli-responsive dynamic transformation and controlled release

Stimulus-responsive delivery systems can use acid-labile bonds, reactive oxygen species (ROS)-sensitive groups, or externally activated materials to alter drug release (Liu et al., 2023b; Liu et al., 2023c). The examples in this subsection include engineered hybrids and chemically modified or carbonized products, not solely reversible assemblies of unmodified plant metabolites. Their introduced carriers and triggers are therefore part of the mechanism and must be considered when interpreting activity (Figure 3).

**FIGURE 3:**
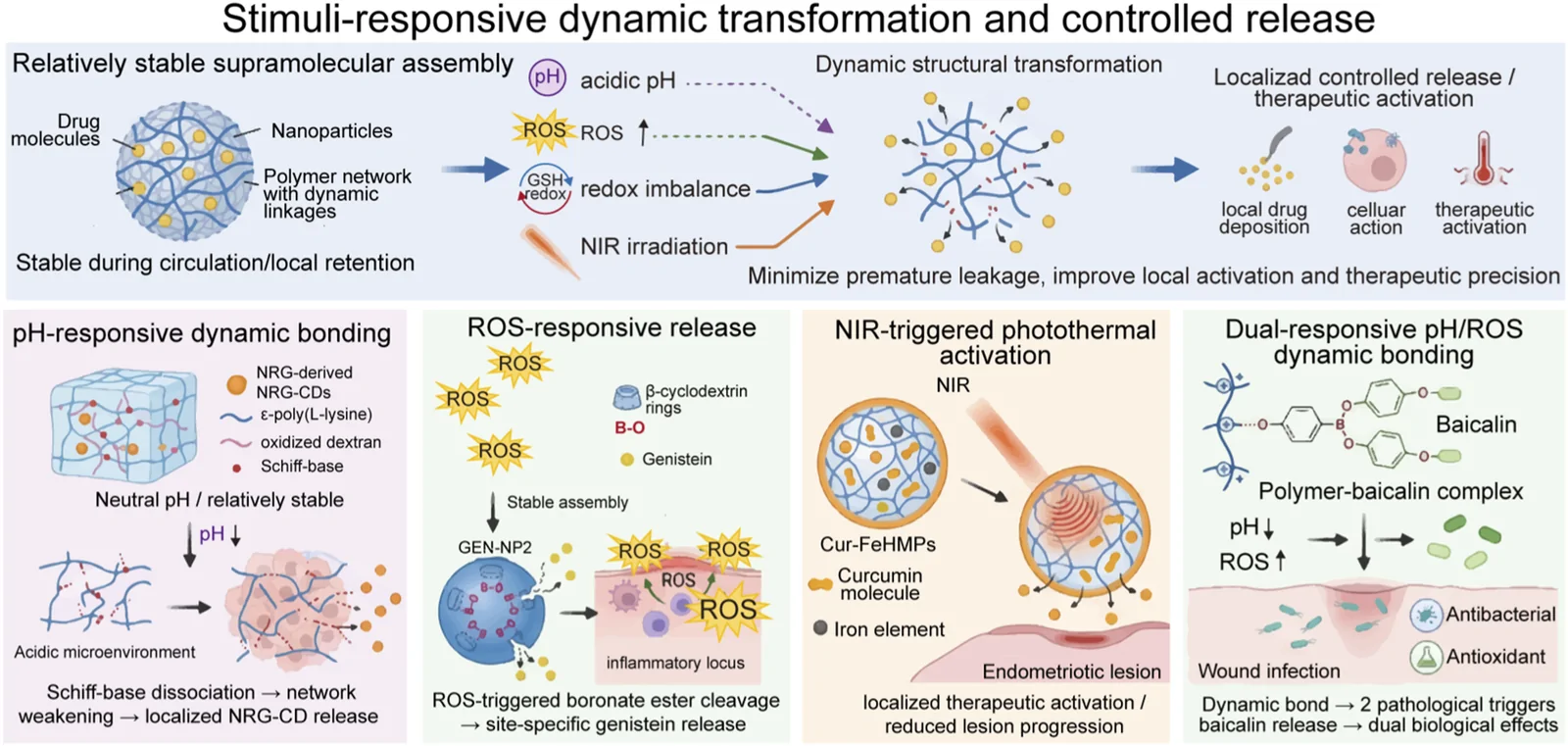
Stimulus-responsive supramolecular materials for controlled release. Upper panel: a relatively stable assembly undergoing stimulus-associated structural change and localized release. Lower panels, from left to right: a pH-responsive hydrogel formed from NRG-CDs through Schiff-base linkages (Yan et al., 2026); ROS-responsive GEN-NP2 containing boronate ester motifs (Fan et al., 2021); NIR-activated Cur-FeHMPs for photothermal treatment of endometriotic lesions (Liu X. et al., 2024); and a polymer-baicalin hydrogel with boronate ester linkages for infected wounds (Yuan et al., 2025). These are engineered, chemically modified or carbonized materials and do not represent native decoction assemblies. Cur-FeHMPs, curcumin-loaded iron-containing hydrogel microspheres; GEN-NP2, genistein-derived ROS-responsive nanoparticles; GSH, glutathione; NIR, near-infrared; NRG-CDs, naringenin-derived carbon dots; ROS, reactive oxygen species.

pH-responsive release is commonly achieved through acid-labile dynamic bonds. Naringenin (NRG)-derived carbon dots (NRG-CDs) integrated with ε-poly (L-lysine) can be crosslinked with oxidized dextran through Schiff-base linkages to form an injectable NRG-CDs-crosslinked oxidized dextran hydrogel (CO-Gel). In acidic tumor microenvironments, reversible Schiff-base dissociation weakens the hydrogel network and enables localized release of NRG-CDs, illustrating how pH-sensitive dynamic covalent bonding connects gel transformation with controlled release (Figure 3) (Yan et al., 2026).

ROS-responsive formulations exploit oxidative stress in inflamed tissues. For example, genistein-derived ROS-responsive nanoparticles (GEN-NP2), constructed from a β-cyclodextrin-based conjugate bearing ROS-cleavable boronate ester motifs, respond to elevated ROS levels by cleaving boronate ester bonds, leading to site-specific genistein release and accumulation at inflamed loci (Figure 3) (Fan et al., 2021). This example demonstrates how oxidative microenvironment signals can be translated into spatially selective drug release.

Curcumin-loaded iron-containing hydrogel microspheres (Cur-FeHMPs) provide a carrier-based photothermal comparator. The formulation uses a crosslinked gelatin methacryloyl matrix containing iron oxide and curcumin, with near-infrared irradiation used to control treatment and release (Liu X. et al., 2024). It should not be described as carrier-free curcumin-iron coordination self-assembly. Its result concerns an engineered composite and does not demonstrate an endogenous stimulus-responsive structure in a decoction (Figure 3).

Boronate ester linkages provide a distinct responsive mechanism. Yuan et al. (2025) combined a cationic polymer bearing phenylboronic acid groups with baicalin to produce a hydrogel for infected wounds after radiotherapy. Its polymer, dynamic bonds and metabolite all contribute to the formulation and must be considered when interpreting release and biological effects (Figure 3). Gallium-quercetin/polylysine particles instead involve metal coordination (Ni et al., 2026); they should not be described as the same boronate-linked material.

For these formulations, responsive release and improved treatment must be separated analytically. Carrier-only controls, free-metabolite controls, and matched formulations with and without the stimulus help identify the contribution of the carrier, irradiation, and release process. Trigger conditions also need comparison with the intended biological environment. A successful engineered response does not establish that an authentic preparation senses the same stimulus or releases its metabolites by the same mechanism.

## 5 Computational interpretation and prospects for predictive modeling

### 5.1 Molecular dynamics simulations and docking-guided mechanistic interpretation

Computational modeling provides a way to examine proposed links between molecular interactions and experimentally observed supramolecular architectures (Jelfs, 2022; Colaço et al., 2024). For TCM-derived SSM, molecular dynamics (MD) simulations can explore assembly trajectories and proposed non-covalent interactions, including π–π stacking, hydrogen bonding, electrostatic attraction, and hydrophobic association. These analyses can help interpret the organization of plant metabolites into NPs, NFs, vesicles, or hydrogel networks within the selected composition, force field, timescale, and boundary conditions. A simulated association supports a mechanistic hypothesis under those assumptions; it does not demonstrate that the same state occurs in a decoction or mediates its biological activity (Figure 4).

**FIGURE 4:**
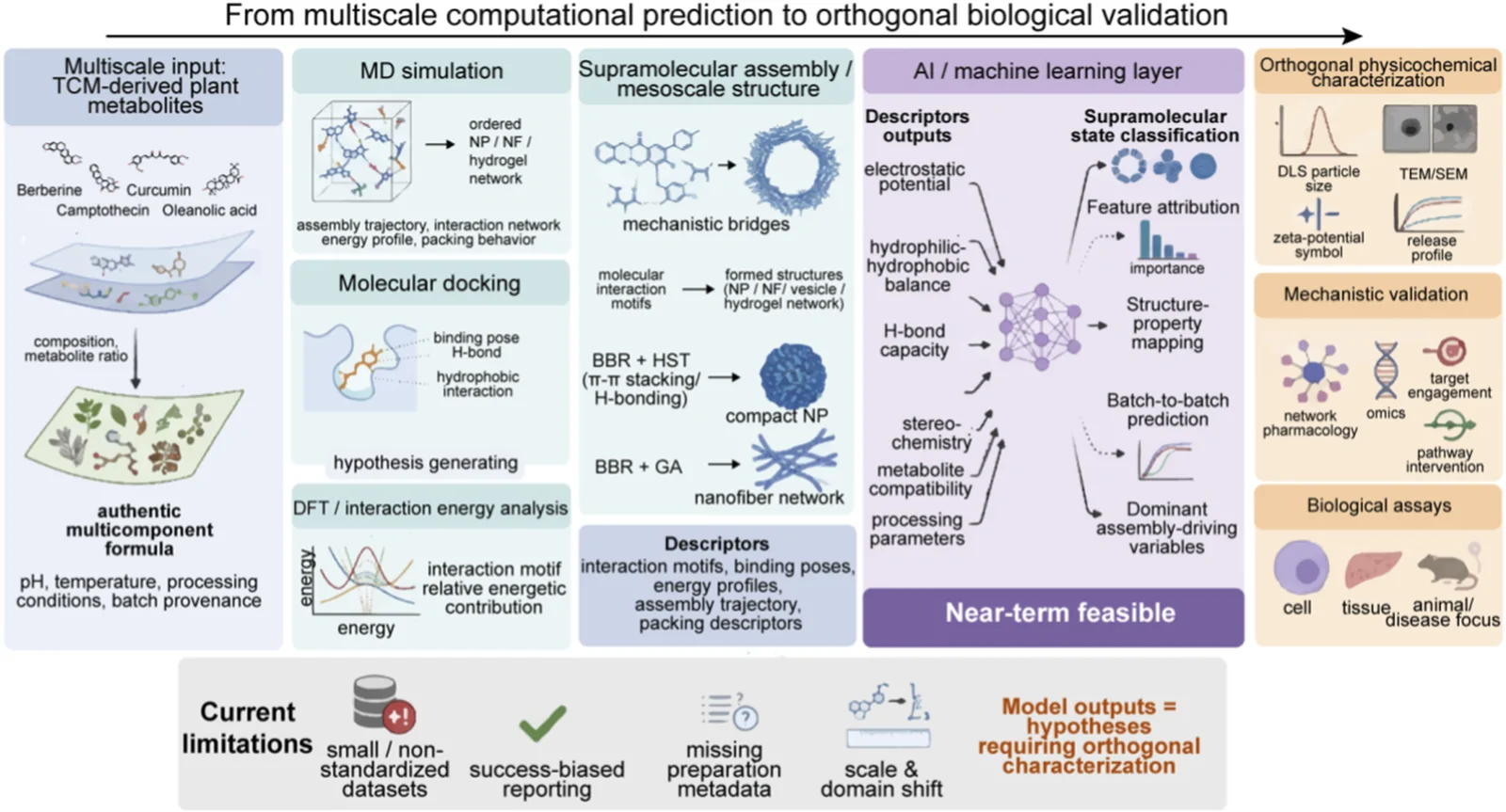
Proposed workflow integrating computational analysis with experimental validation for TCM-derived SSM. Molecular inputs and preparation variables are examined by MD simulation, molecular docking and DFT to generate hypotheses about interaction motifs and assembled structures; the BBR-HST and BBR-GA examples are defined molecular assemblies. The AI layer shows candidate tasks, including state classification, structure-property mapping and batch-to-batch prediction, each of which requires independent validation. Orthogonal physicochemical characterization, mechanistic validation and biological assays are required before model outputs can be interpreted; the lower panel summarizes current limitations. The workflow is a research proposal, not a validated predictor or regulatory standard. AI, artificial intelligence; BBR, berberine; DFT, density functional theory; DLS, dynamic light scattering; GA, glycyrrhizic acid; HST, hesperetin; MD, molecular dynamics; NF, nanofiber; NP, nanoparticle; SEM, scanning electron microscopy; TCM, traditional Chinese medicine; TEM, transmission electron microscopy.

Defined BBR-hesperetin and BBR-GA assemblies illustrate how simulations can be combined with experimental characterization to examine proposed contacts or network formation (Gao et al., 2023; Lei et al., 2025). These are chemically specified models. Agreement between a simulation and selected measurements is informative, but alternative arrangements, sampling limitations, and the effects of omitted solutes must be considered before extending the explanation to an extract.

Docking-guided analysis further connects supramolecular organization with potential biological mechanisms. In Drynariae Rhizoma-derived nanovesicles (RDNVs; botanical drug Drynariae Rhizoma, accepted taxon *Drynaria roosii* Nakaike, syn. *Drynaria fortunei* [Polypodiaceae]), molecular docking suggested that representative bioactive plant metabolites could bind to estrogen receptor alpha (ERα) mainly through hydrogen bonding and hydrophobic interactions, providing a plausible molecular basis for their osteogenic effects (Zhao et al., 2024). However, docking should be regarded as hypothesis-generating rather than definitive evidence. Target engagement assays, pathway intervention, and biological validation remain necessary to confirm the predicted mechanisms.

The methodological limitation is model dependence. MD trajectories require adequate sampling and suitable interaction parameters, while docking scores do not quantify target engagement by an intact assembly. Independent structural measurements and perturbation experiments are needed to test the proposed interpretation. Artificial intelligence (AI) may use such descriptors, but simulated inputs and experimentally measured states must remain distinguishable in the training data.

Density functional theory (DFT) complements docking and MD when the question is the energetic contribution of a weak contact rather than a binding pose or a long trajectory. Combined ligand-based virtual screening and DFT have been used to prioritize small-molecule pairs that subsequently co-assembled into NPs, as in the vinpocetine–lenvatinib example, where hydrogen bonding, van der Waals contacts, and π–π interactions were assigned after experimental assembly (Fang et al., 2025a). An integrated screen of food-derived molecules similarly used 2D/3D matching, docking, DFT, and MD to nominate ursolic acid and 18β-glycyrrhetinic acid, which then formed NPs with antiparasitic activity (Qu et al., 2026). These studies are methodological precedents, not TCM-formula validations; they show that DFT is useful for ranking interaction motifs once candidates exist, not that computation alone establishes self-assembly in a decoction.

Machine-learning models have been trained to predict whether drug–drug pairs form nanomedicines from physicochemical descriptors, with a logistic-regression model reporting 91.89% accuracy on a held-out set of 37 pairs (Zhang et al., 2025). Generative models such as Hydrogel Finder have also proposed self-assembling peptides from non-peptidal small-molecule priors, with a subset confirmed experimentally (Ren et al., 2024). For assemblies of plant metabolites used in TCM, such tools can prioritize metabolites, ratios, or conditions, but domain shift from synthetic or peptide training sets is likely. Model output should be treated as a hypothesis and read together with DFT, MD, and matched experiments, not as independent proof of assembly.

### 5.2 Toward predictive modeling: achievable AI tasks in multicomponent preparations and their current limits

Predicting association among a few defined molecules is a different task from predicting the state of a compositionally variable botanical preparation. Potential applications include classification of measured states, mapping preparation variables to assembly attributes, and prediction across manufacturing batches. These applications are research targets whose feasibility depends on data coverage and independent validation. Inverse design, which starts from a desired property and searches for a formulation, should not be presented as an established capability for authentic multicomponent decoctions on the basis of successful molecular-pair or peptide models (Ren et al., 2024; Zhang et al., 2025).

A testable AI-assisted framework should define connected input, structural-output, and biological-validation layers. The molecular input layer may include electrostatic potential, hydrophilic-hydrophobic balance, hydrogen-bonding capacity, aromaticity, stereochemical configuration, metabolite ratios, and processing parameters. The mesoscale output layer should link these inputs to measurable assembly attributes, including critical micelle concentration, particle size distribution, polydispersity index, morphology, zeta potential, gelation behavior, mechanical properties, release kinetics, and stimuli responsiveness. Graph neural networks, molecular representation learning, and data-driven structure-property models may help capture nonlinear relationships between bioactive plant metabolites and supramolecular architectures (Reiser et al., 2022). Each output must specify measurement time and medium. Model performance should be compared with simpler baselines and assessed on independent batches or preparations within a stated application domain.

Biological interpretation requires an additional validation step. A model that predicts particle size does not thereby predict efficacy, and feature importance does not identify a causal assembly-driving variable. Network pharmacology, docking, or omics associations can suggest experiments but do not replace matched formulation comparisons or direct tests of target engagement. Structural and biological endpoints should therefore be evaluated separately before a combined predictive claim is made.

A practical closed-loop workflow may include four steps: constructing standardized datasets; training models to map molecular and formulation variables to physicochemical descriptors; using optimization algorithms to propose candidate compositions and assembly conditions within the descriptor ranges represented in the training data; and validating predicted assemblies through orthogonal characterization and bioassays. Closed-loop materials discovery strategies, such as Bayesian active learning, provide useful methodological references for this process (Kusne et al., 2020). Both successful and unsuccessful outcomes should be used to update the model. Such work in other materials is a methodological precedent, not validation for TCM decoctions. Prospective comparison with appropriate baselines is needed before using model output to set batch specifications or select a therapeutic preparation (Figure 4).

Several limitations follow from the evidence considered here. A small collection of heterogeneous studies cannot establish generalization to unseen botanical drugs or processing methods. Missing preparation metadata can confound apparent structure-property relationships, and a dataset assembled mainly from successful reports risks selection bias unless failed assemblies and null effects are also sought. State labels must include measurement time and conditions, and train-test separation should prevent related formulations or batches from leaking across sets. Simulations sample chosen models rather than the full chemical diversity and duration of decoction. These issues require explicit evaluation; this narrative review does not quantify their prevalence across the field (Figure 4).

## 6 Biomedical applications and translational potential

### 6.1 Antitumor therapy and tumor microenvironment modulation

Selected engineered formulations containing plant metabolites have been investigated for antitumor delivery, local retention, and combination treatment (Xu et al., 2026). These formulations differ in carrier content, administration route, and mechanism. A reported advantage over a free metabolite therefore needs to be interpreted with the actual dose, comparator, release profile, and tumor model, rather than attributed automatically to supramolecular organization.

Defined sterol-derived assemblies have been investigated as photosensitizing materials (Cheng et al., 2019). By contrast, exosomes loaded with self-assembled nanomicelles constitute a hybrid carrier system, and their barrier transport cannot be attributed to the micelles alone (Cui et al., 2023). Both examples concern engineered interventions, not a demonstration of pre-existing therapeutic particles in an unmodified decoction.

Multicomponent polysaccharide–small molecule–metal nanoassemblies further illustrate how TCM-derived SSM couple molecular recognition with tumor microenvironment (TME)-responsive combination therapy. A pH-responsive nanoassembly composed of *Polygonatum odoratum* (Mill.) Druce [Asparagaceae; Polygonati Odorati Rhizoma] polysaccharide (POP), luteolin (LUT), a pH-responsive bifunctional crosslinker (FH), and manganese ions (Mn^2+^), termed LUT@POP-FH-Mn, undergoes disassembly in the weakly acidic TME. Released LUT induces tumor-cell apoptosis, Mn^2+^ catalyzes Fenton-like reactions to promote lipid peroxidation, and POP together with Mn^2+^ activates dendritic cells, and the combination was reported to produce chemotherapeutic, chemodynamic therapy (CDT) and immunotherapeutic effects (Lin et al., 2026). Similarly, the *Rosa laevigata* Michx. [Rosaceae; Rosae Laevigatae Fructus] polysaccharide (RLP)–isoliquiritigenin (ISL)–iron nanoplatform (ISL@RLP-Fe) integrates polysaccharide immunomodulation, flavonoid bioactivity, and ferric ion-mediated redox cycling, and was reported to amplify ROS generation, lipid peroxidation, and ferroptosis-associated antitumor efficacy (Lin et al., 2025).

Hydrogel-based TCM-derived SSM provide another structure–function strategy by improving local or site-specific drug retention. A *Lycium barbarum* L. [Solanaceae; Lycii Fructus] polysaccharide (LBP)-based oral colon-targeted hydrogel loaded with oxaliplatin, termed Oxa@LBP gel, was reported to achieve sustained oxaliplatin release in the colorectal region while remodeling the immunosuppressive TME through macrophage lipid reprogramming, thereby enhancing treatment of microsatellite-stable colorectal cancer (MSS-CRC) (Zhang et al., 2026). Beyond combination delivery, OA self-assembled micelles were reported to activate the 20S proteasome and promote cancer-cell pyroptosis, whereas elemene hydrogels suppress postoperative tumor recurrence and pulmonary metastasis through local retention and immune microenvironment modulation (Luo et al., 2023; Xian et al., 2024).

These antitumor examples are preclinical formulation studies. They support investigation of particular materials and treatment combinations, not clinical benefit of TCM-derived assemblies as a class. Carrier composition, released metabolites, metal-mediated effects, irradiation, and exposure may contribute alongside assembly. Matched-dose controls and structure-disruption or reconstitution comparisons would help distinguish these contributions. Translation also requires evaluation in clinically relevant settings; efficacy in an animal model is not evidence of improved patient outcomes.

### 6.2 Anti-inflammatory and immunomodulatory therapy

The anti-inflammatory examples below examine defined or engineered formulations in particular disease models. Changes in cytokines, oxidative stress, or tissue injury are biological observations that may accompany altered delivery. To attribute those observations to supramolecular structure, the comparison must also account for metabolite exposure, carrier effects, and the administration route.

For chronic inflammatory diseases, micellar and hydrogel-based TCM-derived SSM can prolong local exposure and regulate immune-cell behavior. Engineered *Panax notoginseng* (Burkill) F.H.Chen [Araliaceae; Notoginseng Radix et Rhizoma] polysaccharide micelles inhibit macrophage polarization and delay rheumatoid arthritis progression through the Janus kinase 2–signal transducer and activator of transcription 3 (JAK2–STAT3) pathway (Yu et al., 2025). Similarly, a sinomenine–GA self-assembled hydrogel enhances the anti-inflammatory effect of sinomenine in rheumatoid arthritis by suppressing nuclear factor-κB (NF-κB)/mitogen-activated protein kinase (MAPK) signaling, reducing pro-inflammatory cytokines, increasing interleukin-10 (IL-10), and inhibiting neutrophil extracellular trap (NET) formation (Jiang et al., 2025a).

Natural-product-derived self-assembled nanomedicines can also regulate mucosal, oxidative, viral, and topical inflammatory injury. Orally deliverable sequence-targeted astaxanthin NPs alleviate colitis, while *L. barbarum* polysaccharide-derived NPs protect against retinal ischemia–reperfusion injury by inhibiting retinal ganglion cell ferroptosis and microglial activation (Chen et al., 2023; Ni et al., 2024). In respiratory inflammation, the baicalin–resveratrol self-assembled nanomedicine (BRN) and the bionic decoction-inspired glabridin–puerarin hydrogel (GLA-PUE@gel) alleviate virus-associated inflammatory injury by suppressing ROS overproduction, restoring cytokine homeostasis, attenuating endoplasmic reticulum (ER) stress, and promoting M2 macrophage polarization (Chang et al., 2025; Qi et al., 2025). In topical inflammation, a GA-containing adhesive nanosystem improves skin retention, lesion penetration, intracellular uptake, and anti-psoriatic efficacy by modulating immune-cell recruitment and inhibiting dendritic-cell maturation (Su et al., 2025).

These studies cannot be treated as evidence of a shared immunomodulatory property of all TCM preparations. A change in a pathway marker or macrophage phenotype is not by itself proof that the assembled state caused the therapeutic effect. The most informative comparisons would hold the chemical dose and route constant, characterize the material during exposure, and test whether the effect persists after selective disruption of the assembly. Chronic and repeated-dose settings also require safety assessment beyond the treatment interval of an individual experiment.

### 6.3 Antimicrobial therapy, wound repair, and tissue regeneration

Antimicrobial delivery and wound repair involve different endpoints, including bacterial burden, tissue exposure, inflammation, and healing. A formulation can improve antibiotic delivery without being antibacterial itself. These distinctions are necessary when evaluating whether an assembly changes microbial interactions, host-cell transport, or both.

Zhang et al. (2020) prepared micelles from the defined saponin GP-682, identified as 3-O-β-D-glucopyranosyl platycodigenin. The study examined increased permeability of host bronchial epithelial cells and improved pulmonary exposure to subsequently administered levofloxacin. The micelles were not simply a levofloxacin-encapsulating preparation, and the relevant membrane effect should not be described as bacterial membrane permeabilization. The combination improved outcomes in a mouse infection model, supporting an adjunctive delivery interpretation rather than antibacterial activity of the micelles alone.

Hydrogel-based TCM-derived SSM further support infected wound repair by integrating antibacterial activity with inflammatory regulation. A natural carrier-free binary small-molecule self-assembled hydrogel was reported to enhance antibacterial effects and promote wound healing by inhibiting bacterial virulence factors and alleviating inflammatory responses (Wang et al., 2023). This case suggests that supramolecular gel networks can act as local therapeutic depots; the relative contributions of molecular self-assembly, tissue retention, bacterial inhibition, and inflammatory microenvironment remodeling require separate evaluation.

The GP-682 example and the hydrogel example address distinct mechanisms and should not be merged into a general claim about antimicrobial self-assemblies. Evidence of host-membrane permeabilization also requires a safety window and assessment of reversibility; evidence of wound closure requires separation of antimicrobial and host-repair effects. Neither result alone establishes clinical efficacy or identifies a stable, active assembly in a traditional decoction.

## 7 Limitations of the evidence and priorities for validation

### 7.1 Preparation source and the limits of extrapolation

The central limitation is the gap between controlled formulations and authentic preparations. Purified-metabolite studies can vary composition and processing deliberately, but decoctions contain changing mixtures of metabolites and macromolecules. Results from one setting cannot be transferred solely because both contain the same named metabolite. Recombined extracts are another distinct setting: freeze-drying, redissolution, heating, and mixing may alter the structures present. The provenance labels in Table 1 therefore describe experimental materials rather than grades of clinical relevance or proof of a common physical state.

Future studies should combine activity-guided fractionation with orthogonal structural characterization, including chromatography–mass spectrometry, nuclear magnetic resonance, dynamic light scattering, cryo-electron microscopy, small-angle scattering, and isothermal titration calorimetry. These approaches can help define metabolite composition, interaction motifs, assembly stoichiometry, particle identity, and time-dependent stability; the limits of state assignment in crude preparations are discussed in Section 3.4 and Table 2. An isolated fraction may not preserve the abundance or structure that existed before separation. Recovery measurements and comparison with the original preparation are therefore required before equating a recovered particle with the pharmacologically decisive entity in the whole formula.

### 7.2 From association to structure-dependent efficacy

An assembled formulation can differ from a free-metabolite control in solubility, dose presentation, release, uptake, and tissue exposure. An efficacy difference consequently supports a formulation-level effect before it establishes that a particular architecture is necessary. The same limitation applies to compatibility interpretations: a defined molecular pair can support a plausible interaction without demonstrating the causal mechanism of a multicomponent prescription.

Future studies should introduce systematic decoupling strategies to separate composition-dependent effects from structure-dependent effects. Useful comparisons include free metabolites, physical mixtures, disrupted assemblies, reassembled structures, carrier-only preparations where relevant, and size-, charge-, or rigidity-matched controls with identical chemical composition. Biological assays should then be coupled with quantitative structural characterization to test whether morphology, surface properties, mechanical behavior, or stimuli-responsive transformation contributes to cellular uptake, tissue penetration, immune regulation, controlled release, or therapeutic efficacy. Chemical composition, dose, medium and processing should be matched sufficiently to isolate the intended variable. Disruption or depletion can itself change chemistry, making recovery and add-back controls essential.

### 7.3 Methodological comparability and candidate quality attributes

Cross-study comparison is limited when materials are characterized under different concentrations, solvents, pH values, temperatures, or observation times. For botanical preparations, identity and provenance of the raw materials, extraction ratio, heating history, mixing order, filtration, storage, and sampling should accompany the structural and biological data. Best-practice recommendations for phytopharmacological research, including the ConPhyMP guidelines for extract characterization, provide a practical reference for such reporting (Heinrich et al., 2020; Heinrich et al., 2022). Table 3 applies these items to the botanical drug preparations discussed here; authentication and voucher specimens were reported for only one of the five preparations, and none reported the legal basis for collecting the plant material. Missing information prevents assessment of comparability and reproducibility, but should be recorded study by study rather than converted into an unsupported estimate of poor reporting across the field.

Particle size distribution, composition, morphology, release behavior, and gel mechanics are possible quality measurements, not automatically validated critical quality attributes (CQAs). Within a Quality by Design (QbD) approach, a candidate measurement must be linked to a relevant product outcome and shown to be reproducible and sensitive to meaningful batch variation (International Conference on Harmonisation, 2009). Raw-material attributes and process parameters are inputs to that evaluation and should not be conflated with attributes of the finished product. No universal acceptance limit can be inferred from the heterogeneous examples reviewed here.

Regulatory assessment is product- and route-specific. A purified metabolite formulation, a complex botanical preparation, and a carbonized or polymer-based hybrid may raise different questions of identity, manufacturing, safety, and performance. Guidance on products containing nanomaterials provides a relevant development context (U.S. Food and Drug Administration, 2022), but the framework proposed here is not a validated regulatory pathway or evidence that an assembly-based quality system has already improved a marketed product.

### 7.4 *In vivo* integrity, safety and clinical translation

Pharmacokinetic and biosafety evaluation is an important translational checkpoint for TCM-derived SSM. Assemblies may undergo protein-corona formation, dilution- or enzyme-induced disassembly, structural remodeling, and metabolite exchange after entering plasma, mucus, interstitial fluid, or inflammatory exudates. These dynamic transformations can alter biological identity, circulation time, tissue accessibility, cellular uptake, metabolism, excretion, and therapeutic exposure (Mahmoudi et al., 2023). Future studies should distinguish the *in vivo* fate of free metabolites, intact assemblies, disassembled material, and newly formed biomolecular complexes. Total drug concentration or fluorescence localization does not by itself distinguish an intact assembly from released metabolites or detached labels.

Translational evaluation should move beyond measuring total drug concentration and integrate pharmacokinetics with structural-state analysis. For TCM-derived SSM, route-specific assessment is particularly important because oral, injectable, topical, and local administration may expose assemblies to different dilution conditions, enzymes, proteins, immune cells, mucus barriers, and tissue microenvironments. Evaluation of biodistribution, metabolism, excretion, immune responses, organ toxicity, and repeated-administration safety should match the intended route and duration of treatment (Cabral et al., 2024). Exposure measurements should distinguish free and associated metabolite fractions where technically feasible. A plant origin does not establish biocompatibility, and safety of an isolated metabolite cannot simply be transferred to a nanoparticle, chemically modified derivative, or carbonized product.

The preclinical examples discussed here do not establish patient benefit attributable specifically to supramolecular organization. Clinical translation requires defined manufacturing, reproducible product attributes, suitable toxicology and comparative clinical evaluation (Joyce et al., 2024). Therapeutic conclusions should remain specific to the experimental setting, comparator and endpoint studied. Clinical evidence would need a dedicated synthesis beyond the scope of this narrative review.

### 7.5 Selection and reporting limitations of this review

An example-based narrative synthesis is vulnerable to selective coverage and to greater visibility of successful formulations. This review has not measured publication bias or quantified how often assembly attempts fail. Future evidence collections should seek unsuccessful preparations, null biological comparisons, and independent replications, and record uncertainties or unavailable methodological details explicitly. Prospective data collection with common definitions would provide a stronger basis for comparing materials and developing models than treating a collection of positive case reports as a representative training set.

## 8 Conclusion

Selected botanical drug preparations and engineered plant-metabolite formulations provide evidence that physical organization can influence measured properties and preclinical responses. The strength of that evidence depends on the material tested, the structural measurements, and the biological comparison. Assemblies isolated from decoctions, recombined extracts, purified models, and carrier-containing hybrids answer different questions and should remain distinguishable in both tables and narrative conclusions. The value of a supramolecular perspective is to make composition, organization, and experimental conditions jointly testable, rather than to assign a new universal property to TCM.

The priorities are to identify relevant assembled species without uncontrolled separation artifacts, distinguish structural effects from chemical dose and exposure, and determine whether assemblies persist in the intended biological setting. Quality control and predictive design remain prospective uses of such evidence. They require reproducible measurements, independent validation, and an explicit domain of application. The framework presented here supports that evaluation while keeping claims proportional to what the cited studies demonstrate.

## Glossary

AI: artificial intelligence
BBR: berberine
BPNs: boronate–phenolic networks
BRN: baicalin–resveratrol self-assembled nanomedicine
CDT: chemodynamic therapy
CMC: critical micelle concentration
CO-Gel: NRG-CDs-crosslinked oxidized dextran hydrogel
CQA: critical quality attribute
CQAs: critical quality attributes
CR: Coptidis Rhizoma
Cur-FeHMPs: curcumin-loaded iron-containing hydrogel microspheres
DFT: Density functional theory
DPD: dissipative particle dynamics
ECM: extracellular matrix
ER: endoplasmic reticulum
ERα: estrogen receptor alpha
FH: pH-responsive bifunctional crosslinker
GA: glycyrrhizic acid
GAO: glycyrrhizic acid–oxymatrine bioactive ionic liquid
GelMA: gelatin methacryloyl
GEN-NP2: genistein-derived reactive oxygen species-responsive nanoparticles
GLA-PUE@gel: glabridin–puerarin self-assembled hydrogel
GNN: graph neural network
GSH: Glutathione
GP-682: 3-O-β-D-glucopyranosyl platycodigenin
IL-10: interleukin-10
ISL: isoliquiritigenin
ISL@RLP-Fe: Rosa laevigata polysaccharide–isoliquiritigenin–iron nanoplatform
JAK2–STAT3: Janus kinase 2–signal transducer and activator of transcription 3
LBP: Lycium barbarum polysaccharide
LUT: luteolin
LUT@POP-FH-Mn: luteolin–Polygonatum odoratum polysaccharide–pH-responsive bifunctional crosslinker–manganese nanoassembly
MAPK: mitogen-activated protein kinase
MASH: metabolic dysfunction-associated steatohepatitis
MD: molecular dynamics
MPNs: metal–phenolic networks
MSS-CRC: microsatellite-stable colorectal cancer
NET: neutrophil extracellular trap
NF-κB: nuclear factor-κB
NFs: nanofibers
NIR: near-infrared
NPs: nanoparticles
NRG: naringenin
NRG-CDs: naringenin-derived carbon dots
OA: oleanolic acid
OA-NG: oleanolic acid nanogel
OA-NGs: oleanolic acid nanogels
OA-NP: oleanolic acid nanoparticle
OA-NPs: oleanolic acid nanoparticles
OMT: oxymatrine
Oxa: oxaliplatin
Oxa@LBP gel: oxaliplatin-loaded Lycium barbarum polysaccharide hydrogel
PAL-4: palmitoyl pentapeptide-4
PDI: polydispersity index
PGG: 1,2,3,4,6-penta-O-galloyl-D-glucose
POP: Polygonatum odoratum polysaccharide
PUE: puerarin
QbD: Quality by Design
RDNVs: Rhizoma Drynariae-derived nanovesicles
RLP: Rosa laevigata polysaccharide
ROS: reactive oxygen species
SSM: supramolecular soft matter
TCM: traditional Chinese medicine
TCM-derived SSM: traditional Chinese medicine-derived supramolecular soft matter
TME: tumor microenvironment
ZR1: Zingibroside R1
